# Genetic and Morphological Diversity Assessment of Five Kalanchoe Genotypes by SCoT, ISSR and RAPD-PCR Markers

**DOI:** 10.3390/plants11131722

**Published:** 2022-06-29

**Authors:** Jameel M. Al-Khayri, Ehab M. B. Mahdy, Heba S. A. Taha, Ahmed S. Eldomiaty, Mohamed A. Abd-Elfattah, Arafat Abdel Hamed Abdel Latef, Adel A. Rezk, Wael F. Shehata, Mustafa I. Almaghasla, Tarek A. Shalaby, Muhammad N. Sattar, Hesham S. Ghazzawy, Mohamed F. Awad, Khalid M. Alali, Shri Mohan Jain, Abdallah A. Hassanin

**Affiliations:** 1 Department of Agricultural Biotechnology, College of Agriculture and Food Sciences, King Faisal University, Al-Ahsa 31982, Saudi Arabia; arazk@kfu.edu.sa (A.A.R.); wshehata@kfu.edu.sa (W.F.S.); kalali@kfu.edu.sa (K.M.A.); 2 National Gene Bank (NGB), Agricultural Research Centre (ARC), Giza 12613, Egypt; ehab.mahdy@arc.sci.eg; 3 Genetics Department, Faculty of Agriculture, Zagazig University, Zagazig 44511, Egypt; hebasayedtaha@gmail.com (H.S.A.T.); a.salah8373@gmail.com (A.S.E.); 4 Pomology Department, Faculty of Agriculture, Cairo University, Giza 12613, Egypt; dr.mohamed.alaa@cu.edu.eg; 5 Department of Botany and Microbiology, Faculty of Science, South Valley University, Qena 83523, Egypt; 6 Department of Arid Land Agriculture, College of Agriculture and Food Sciences, King Faisal University, P.O. Box 420, Al-Ahsa 31982, Saudi Arabia; malmghaslah@kfu.edu.sa (M.I.A.); tshalaby@kfu.edu.sa (T.A.S.); 7 Plant Pests, and Diseases Unit, College of Agriculture and Food Sciences, King Faisal University, P.O. Box 420, Al-Ahsa 31982, Saudi Arabia; 8 Horticulture Department, Faculty of Agriculture, Kafrelsheikh University, Kafr El-Sheikh 33516, Egypt; 9 Central Laboratories, King Faisal University, P.O. Box 420, Al-Ahsa 31982, Saudi Arabia; mnsattar@kfu.edu.sa; 10 Date Palm Research Center of Excellence, King Faisal University, Al-Ahsa 31982, Saudi Arabia; hghazzawy@kfu.edu.sa; 11 Department of Biology, College of Science, Taif University, P.O. Box 11099, Taif 21944, Saudi Arabia; m.fadl@tu.edu.sa; 12 Department of Agricultural Sciences, University of Helsinki, 00014 Helsinki, Finland; jain.mohan70@gmail.com

**Keywords:** genetic polymorphism, diversity assessment, molecular markers, SCoT, ISSR, RAPD

## Abstract

Determining the appropriate parents for breeding programs is the most important decision that plant breeders must make to maximize the genetic variability and produce excellent recombinant genotypes. Several methods are used to identify genotypes with desirable phenotypic features for breeding experiments. In this study, five kalanchoe genotypes were morphologically characterized by assessing plant height, number of inflorescences, number of flowers, flower length, flower diameter and number of petals. The analysis showed the distinction of yellow kalanchoe in the plant height trait, while the orange kalanchoe was distinguished in the number of inflorescences, the number of flowers and flower length traits, whereas the violet kalanchoe possessed the largest flower diameter and the highest number of petals. The molecular profiling was performed by random amplified polymorphism DNA (RAPD), inter-simple sequence repeats (ISSR) and start codon targeted (SCoT)-*polymerase chain reaction* *(PCR)* tools. Genomic DNA was extracted from young leaves and the PCR reactions were performed using ten primers for each SCoT, ISSR and RAPD marker. Only four out of ten primers showed amplicon profiles in all PCR markers. A total of 70 bands were generated by SCoT, ISSR and RAPD-PCR with 35 polymorphic bands and 35 monomorphic bands. The total number of bands of RAPD, ISSR and SCoT was 15, 17 and 38, respectively. The polymorphism percentages achieved by RAPD, ISSR and SCoT were 60.25%, 15% and 57%, respectively. The cluster analysis based on morphological data revealed two clusters. Cluster I consisted of violet and orange kalanchoe, and cluster II comprised red, yellow and purple kalanchoe. Whereas the cluster analysis based on molecular data revealed three clusters. Cluster I included only yellow kalanchoe, cluster II comprised orange and violet kalanchoe while cluster III comprised red, and purple kalanchoe. The study concluded that orange, violet and yellow kalanchoe are distinguished parents for breeding economically valued traits in kalanchoe. Also, the study concluded that SCoT and RAPD markers reproduced reliable banding patterns to assess the genetic polymorphism among kalanchoe genotypes that consider the basis stone for genetic improvements in ornamental plants.

## 1. Introduction

Kalanchoe is a medicinal plant largely used in folk medicine for the treatment of kidney stones, gastric ulcer, pulmonary infection and rheumatoid arthritis [1] and is grown commercially as a flowering potted plant [2]. The kalanchoe genus comprises 125 species of Crassulaceae succulent plants [3]. The majority of the kalanchoe genotypes are native to Madagascar and tropical Africa, and many of them are popular due to their growing indoors [4]. Kalanchoes are very low-maintenance houseplants, however, they require direct sunlight. They can also endure bright indirect light and only need to be watered when completely dry. Leaf or stem cuttings can be used to reproduce all species. Kalanchoe genotypes are mostly perennial herbaceous plants, with a few shrubs and annuals. The thick leaves are waxy or hairy and come in a variety of shapes. They are frequently borne on the stems in opposite directions. From the plant’s base or along the leaf margins, several species develop clonal plantlets [3].

The species of kalanchoe play an important role in scientific research of genetic diversity and evolutionary aspects of plants. Plant diversity has captivated humans throughout history, owing to the enormous variation in molecular and morphological features found in nature. The naturally occurring variations among plant species provide a familiar environment for evolution by natural selection, plant taxonomy and phylogeny are based on genetic diversity [5,6,7].

In the field of ornamental plant improvement, distant hybridizations are still a common method of promoting genetic diversity in plants. More than 100 plant species have derived from this variation in breeding and genetic improvement programs [6,8,9] by applying direct selection for specific traits. Understanding the genetic basis and molecular profiles of all this naturally occurring diversity is one of the fundamental challenges of modern biology.

Several studies discussed the genetic diversity of various ornamental plants such as *Allium* species [10], ornamental *Coffea Arabica* plants [11], ornamental pomegranates (*Punica granatum* L.) [12], ornamental pepper plants [13] and *Dianthus* [14].

Conventional DNA markers have numerous applications in determining genetic diversity in plants. These markers include inter simple sequence repeat (ISSR) markers [15], sequence-related amplified polymorphism (SRAP) markers [16], and simple sequence repeat (SSR) markers [16]. Recently, new promising techniques have emerged. Start Codon Targeted (SCoT) is a dominant and reproducible marker that is based on the short conserved region in plant genes surrounding the ATG start codon [17]. To obtain more information about the morphological and molecular profiles of some kalanchoe genotypes and their phylogenetic relationship, we have applied phenotypic characterization and molecular profiling; SCoT, ISSR and RAPD-PCR analyses to compare five selected kalanchoe genotypes.

This investigation was performed to assess the genetic diversity among five kalanchoe genotypes by combining morphological characterization with the RAPD, ISSR and SCoT molecular markers and phylogeny analyses.

## 2. Results

### 2.1. Morphological Polymorphism

**Figure 1:**
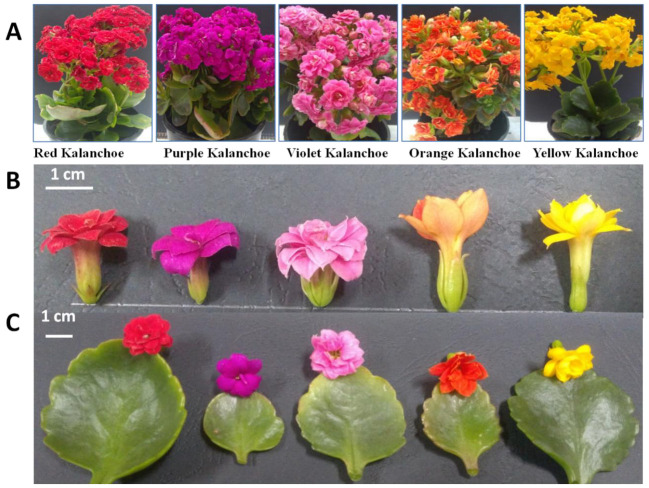
Morphological characterization of five kalanchoe (*Kalanchoe blossfeldiana*) genotypes. (**A**) Different kalanchoe plants with various colors illustrate plant architecture. (**B**) Flower colors polymorphism. (**C**) Difference in leaf shape and size.

**Figure 2:**
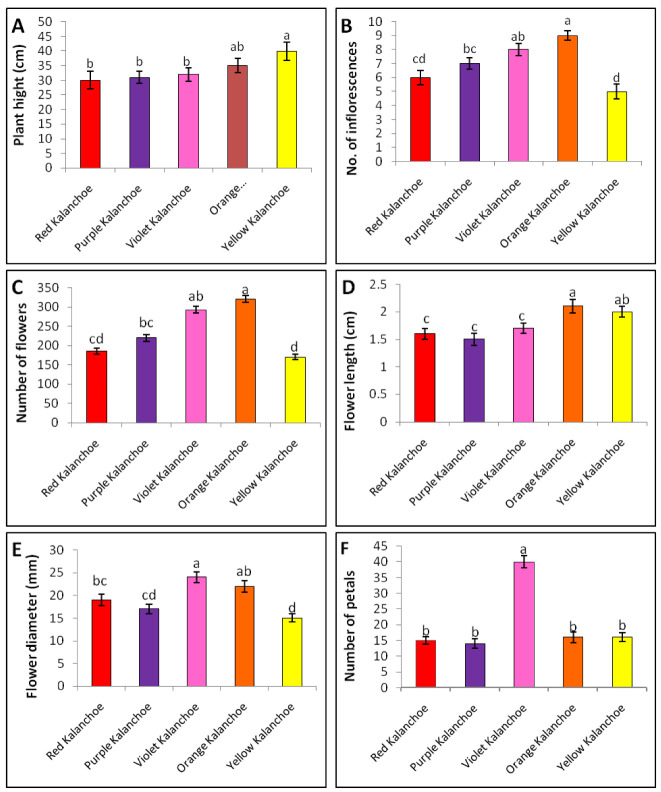
Selected characteristics of five kalanchoe genotypes; each presented value is means ± standard error, and the different letters mean that values are significantly different (*p* ≤ 0.05) according to Fisher’sLSD_0.05_. (**A**) plant height (**B**) Number of inflorescences. (**C**) Number of flowers (**D**) Flower length (**E**) Flower diameter (**F**) Number of petals. Note: (**A**–**C**) Average of 10 plants (**D**–**F**) Average of 50 flowers/plant.

Five kalanchoe genotypes were maintained under greenhouse conditions and assessed for morphological features; number of petals, flower length, number of flowers, flower diameter, number of inflorescences and plant height (Figure 1 and Figure 2) which have economic importance in ornamental plants.

The yellow kalanchoe genotype showed the highest plant height (40 cm), while the orange kalanchoe genotype got the lion’s share due to recording the highest number of inflorescences (9 inflorescences plant^−1^), number of flowers (321 flowers plant^−1^) and flower length (2.1 cm). The violet genotype recorded the highest number of petals (40 petals flower^−1^).

Statistical analysis revealed significant and non-significant differences among the five kalanchoe genotypes in morphological characteristics based on the least significant difference (LSD) values. A significant difference was noticed between the yellow kalanchoe genotype and the remaining genotypes, except for the orange kalanchoe in plant height character (Figure 2A). The analysis showed a significant difference in the number of inflorescences between orange kalanchoe genotype and the rest of the genotypes except the violet genotype, a significant difference also appeared between each of red and yellow genotypes and violet and purple kalanchoe genotypes (Figure 2B). Two insignificant differences; the first one appeared between purple and violet kalanchoe and the second one between red and yellow kalanchoe (Figure 2B). The number of flowers showed a significant difference between orange kalanchoe genotype and the rest of the genotypes except the violet genotype exactly as the statistical profile appeared in the number of inflorescences (Figure 2C).

Flower length (cm) was significantly different among both orange and yellow genotypes and the rest genotypes, while violet, purple and red genotypes were insignificantly different from each other (Figure 2D). Flower diameter showed an insignificant difference between the orange and the violet kalanchoe genotypes, while they presented significant differences with the rest of the genotypes (Figure 2E). Orange and red genotypes were insignificantly different from each other, while they recorded significant differences with purple and yellow genotypes. The yellow genotype presented a significant difference with all genotypes in flower diameter character (Figure 2E). Finally, the number of petals showed a significant difference between the violet genotype and the rest of the genotypes which presented insignificant differences from each other’s (Figure 2F).

### 2.2. Molecular Polymorphism Analyses

**Figure 3:**
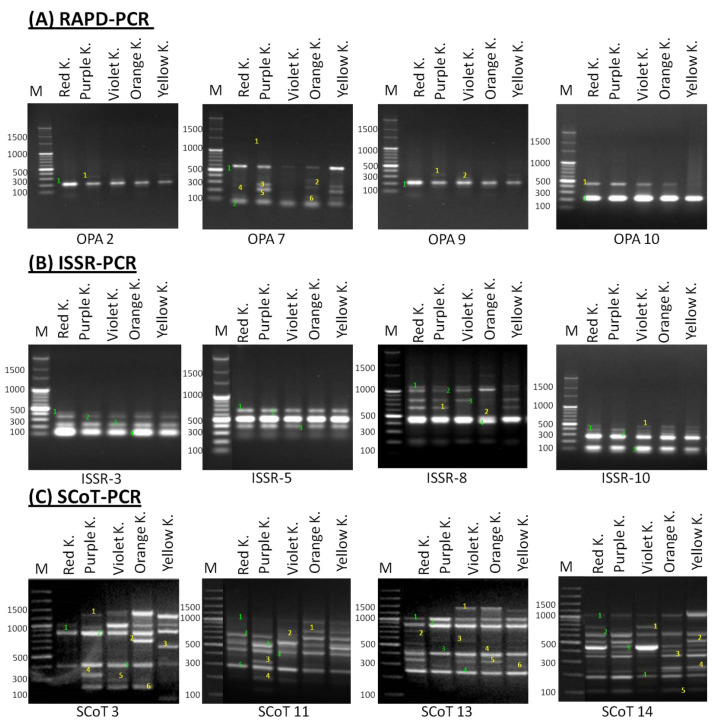
DNA fragment patterns of RAPD, ISSR and SCoT-PCR amplification of five kalanchoe genotypes. (**A**) RAPD-PCR amplification using primers OPA 2, OPA 7, OPA 9 and OPA 10, respectively. (**B**) ISSR-PCR amplification using primers ISSR-3, ISSR-5, ISSR-8 and ISSR-10, respectively. (**C**) SCoT-PCR amplification using primers SCoT3, SCoT11, SCoT13 and SCoT14, respectively. M = 100 bp Plus DNA Ladder. The monomorphic loci are presented in green numbers and the polymorphic loci are presented in yellow numbers.

Genetic polymorphism analysis of kalanchoe genotypes was conducted by RAPD, ISSR and SCoT-PCR amplifications using 30 arbitrary primers (10 primers for each PCR reaction type) (Please see materials and methods section) to examine the molecular polymorphism among kalanchoe genotypes. Finally, only four primers for each reaction produced reliable polymorphic banding profiles within all the studied genotypes (Table 1 and Figure 3). The reactions of PCR for the three techniques produced 70 loci, 35 of which were polymorphic, while 35 were monomorphic. The total number of amplified loci for RAPD, ISSR and SCoT-PCR was 17, 17 and 38, respectively (Table 1). The polymorphism revealed by RAPD-PCR ranged from 50% to 70% while the polymorphism revealed by ISSR-PCR ranged between zero % to 33% and the polymorphism produced by SCoT-PCR ranged from 44% to 66% (Table 1).

### 2.3. Phylogeny Analyses

**Figure 4:**
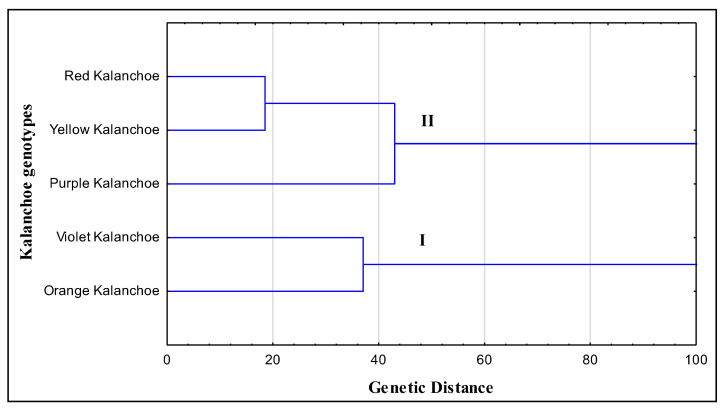
Phylogenetic tree of five kalanchoe genotypes revealed by the weighted pair group method using arithmetic average (WPGMA) method based on morphological features.

**Figure 5:**
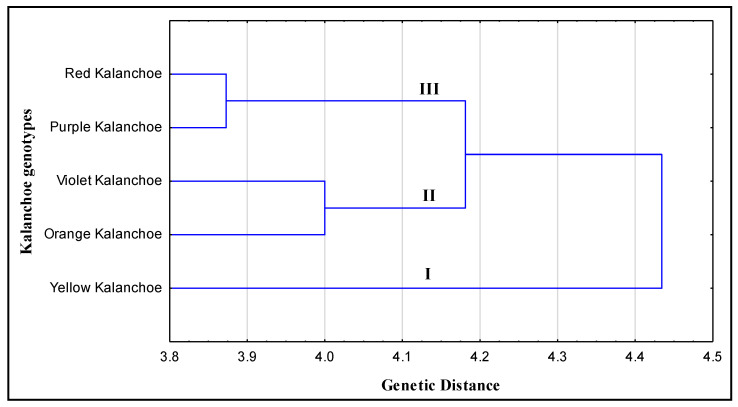
Phylogenetic tree of five kalanchoe genotypes revealed by the weighted pair group method using arithmetic average (WPGMA) method based on SCoT, ISSR and RAPD banding patterns.

Phylogenetic relationships among the five kalanchoe genotypes were inferred based on the data recorded from morphological criteria. The clustering analysis grouped the five kalanchoe genotypes into two groups (I, II) (Figure 4). Cluster I comprised the violet and the orange kalanchoe genotypes and cluster II included red, yellow and purple kalanchoe genotypes. On the other hand, the phylogenetic relationship was determined among the kalanchoe genotypes based on the banding profiles revealed by RAPD, ISSR and SCoT-PCR. Phylogenetic analysis (Figure 5) divided the five kalanchoe genotypes into three clusters according to the data scored from the molecular analysis. Yellow kalanchoe independently formed cluster Ι. The analysis grouped both violet and orange kalanchoe in cluster II. Cluster III included purple and Red kalanchoe genotypes. The only difference between the clustering of kalanchoe genotypes based on molecular and morphological attributes is that the yellow kalanchoe grouped in an independent group based on the molecular profile (Figure 5).

## 3. Discussion

The success of breeding programs is determined by the accurate selection of parents. At this point, breeders begin their search for a specific plant genotype that will meet market expectations. Even while recombination may play role in expanding the polymorphism among segregating populations, the ability of two parents to combine their progeny and their excellent performance in agronomic variables will identify whether the progeny will be successful elite lines [18,19]. Given the scarcity of knowledge on combining abilities, investigations that highlight genotype correlations will be critical sources for investigations, assisting breeders to select parents for breeding experiments. As a result, morphological and DNA marker characterizations, as well as multivariate statistical analyses, will be critical components in improving our capability to select the best parents for crosses [18].

In this investigation, five kalanchoe genotypes were characterized regarding their morphological and molecular features. The current results of morphological characterization revealed that some kalanchoe genotypes had advantages in some traits, and this can be considered as a cornerstone in breeding and genetic improvement of kalanchoe. The orange kalanchoe offered advantages in its number of inflorescences, number of flowers and flower length, which are important traits in the characterization of ornamental plants. Hence, orange kalanchoe genotypes may be used as parents in breeding programs for these parameters.

In the same context, the yellow kalanchoe presented advantages in both plant height and flower length, also the violet kalanchoe showed the highest score in flower diameter and number of petals which are considered the most important features in the ornamental field. For successful Kalanchoe breeding programs, it is important to completely characterize the morphological traits, especially those that have economic importance such as number of inflorescences, number of flowers and flower length. Achieving desired breeding results requires correct parent selection and understanding of the genetic distance either based on morphological features or molecular profiling and understanding the inheritance of the desired traits [20]. This study clarified that *Kalanchoe* genotypes are morphologically diverse and hence are great candidates for intraspecific hybridization to produce new lines with favorable features.

The current study emphasized the potential importance of DNA polymorphism detection for plant breeding programs and genetic improvement. Plant genetic diversity can be studied and detected with the help of molecular markers [21]. For genetic diversity studies in plants, a variety of molecular markers are used, including RAPD [22,23], ISSR [24], amplified fragment length polymorphism (AFLP) [25,26] and SCoT [27].

The molecular markers (RAPD and SCoT) produced reliable polymorphic banding patterns enabling the determination of genetic polymorphism among kalanchoe genotypes. The ISSR produced a low polymorphism ratio compared to both RAPD and SCoT markers. The RAPD and SCoT-PCR markers used in this study may be considered as more efficient in identifying the genetic polymorphism based on their polymorphic banding profiles than ISSR-PCR which produced a low polymorphism percentage. This may provide important clues in distinguishing the relationship among kalanchoe genotypes. These results are similar to those reported by Collard and Mackill [17], they found that results of amplification yield using SCoT technique were more reproducible than other molecular markers results in rice. Furthermore, genetic polymorphisms produced by SCoT marker were better in determining the relationship among mango cultivars than ISSR markers [28]. In the same context, an investigation on potato indicated the greater effectiveness of SCoT markers in determining somaclonal variation compared to ISSR and RAPD markers [29].

Based on the results obtained from morphological data and molecular profiles of the kalanchoe genotypes, we generated two phylogenetic trees. Phylogeny and alignment are important analyses to determine the genetic diversity among different species [6,30,31,32,33,34,35]. The phylogeny analysis showed similar results in clustering of the genotypes except the analysis based on molecular banding patterns separated the yellow kalanchoe in an independent cluster, while the other four genotypes were grouped in two clusters similarly in both analyses based on morphological and molecular profiles. It is clear from the cluster analysis that genotypes of the same cluster may have common ancestors. So we combined results from both molecular and morphological analyses to precisely interpret and shed light on the genetic relationship among kalanchoe genotypes that can be considered a cornerstone for genetic improvement. Based on the genetic relationship, suitable parents can be selected for hybridization experiments to achieve desired heterosis effects. Generally, the number of data derived from morphological observations is significantly fewer than that derived from genetic markers, resulting in a bias toward the molecular analyses’ outcome [18]. When morphological criteria and molecular markers were examined in wheat, three out of four clusters in the molecular analysis were consistent with the distance indicated by phenotypic features [36].

Despite the current study may support the conventional breeding methods for genetic improvement of kalanchoe genotypes, however recent genetic approaches such as genetic engineering [37] or genome editing approaches [38,39] could be efficiently used in kalanchoe breeding and improvement for different desirable features.

## 4. Materials and Methods

### 4.1. Plant Materials

The five genotypes of kalanchoe used in this study were obtained and classified by a plant taxonomist in the Faculty of Science. The plants were kept in a controlled greenhouse environment with natural light, at a temperature of 15 °C at night and 25 °C during the day.

### 4.2. Morphological Polymorphism

Morphological data; plant height, number of inflorescences, number of flowers, flower length, flower diameter and number of petals were collected. Plant height was recorded on the day when the first flower bloomed. Flower diameter, floral length, and the number of petals were recorded at the sticky stage of stigma [40]. On the day that the first wilted flower was spotted, the number of blooms and inflorescences were counted.

### 4.3. Genomic DNA Extraction

The genomic DNA was extracted from 5 g of sterilized (0.05% Clorox) young leaf samples using the cetyltrimethylammonium bromide (CTAB) method [41]. A Nano Drop 2000 (Thermo Scientific™, Waltham, MA, USA) was used for measuring extracted DNA concentrations in the samples; electrophoresis on a 1% agarose gel was performed to verify the quality and quantity of extracted DNA. The concentrations of DNA were set up to 50 ng·μL^–1^ and DNA was stored at –20 °C for the next amplification experiments.

### 4.4. Random Amplified Polymorphism DNA (RAPD-PCR)

PCR amplification was performed by ten primers as shown in Table 2 according to Williams, Kubelik, Livak, Rafalski and Tingey [22]. The RAPD-PCR amplification reaction was conducted in a 25 μL reaction mixture including 15 μLof 2x fidelity Taq PCR Master Mix (USB Corporation, Cleveland, OH), 2 μLdNTPs (200 μM), 1.5 mM MgCl_2_ (25 mM), 1 μM of each primer (10 pmol) and 2 μL of genomic DNA (20 ng/μL). The final volumes were adjusted with sterile distilled water up to 25 μL. The RAPD-PCR-based amplification was performed using a 96 well plate thermal cycler (Applied Biosystem) as the following: 95 °C for 1 min for initial denaturation, followed by 40 cycles of 95 °C for 30 s, 1 min at the annealing of each primer and 72 °C for 1 min for extension and the final extensions were done at 72 °C for 5 min.

### 4.5. Inter-Simple Sequence Repeats (ISSR-PCR)

ISSR-PCR-based reaction was performed to detect the polymorphism among kalanchoe genotypes using the primers presented in Table 2. The reaction was conducted according to procedures described by Moreno, Martín and Ortiz [42]. The ISSR-PCR-based amplifications were conducted in a 25 μL reaction mixture containing 20 ng/μL of template DNA, 2 μL 5× buffer; 2 μL MgCl_2_ (25 mM), 2 μL dNTPs (200 μM), 2 μL Primer (10 pmol) and 1 Unit Taq DNA polymerase (Promega). The conditions of ISSR-PCR amplifications were used in an initial denaturation of 94 °C for 5 min followed by 35 cycles of 94 °C for 1 min, the annealing temperature for the various primers for 1 min, 72 °C for 1 min (extension) and final extension at 72 °C for 5 min.

### 4.6. Start Codon Targeted (SCoT) Amplification

The SCoT primers (Table 2) were chosen according to [17]. The SCoT-PCR-based reactions were conducted in a 25 μL reaction mixture containing 25 ng template DNA, 0.2 μM dNTPs, 1.5 µM of each primer, 1.5 mM MgCl_2_, 2 μL 5× buffer and 1 Unit of Taq polymerase (Promega). The PCR amplification was set up for the initial denaturation at 94 °C for 5 min, followed by 35 cycles, each cycle comprised of 94 °C for 1 min, 53 °C for 1 min, then 72 °C for 120s, and the final extensions were done at 72 °C for 7 min.

### 4.7. Gel Electrophoresis

1.5% agarose gel electrophoresis in TBE buffer was used to separate the results of the RAPD, ISSR, and SCoT reactions according to [43]. The size of DNA bands on the gel was calculated using 100 bp DNA ladder (GeneRuler 100 bp Plus DNA Ladder, Thermo Fischer Scientific, USA). Ethidium bromide (MP Biomedicals, Goddard Irvine, CA, USA) was used to stain the agarose gel that was further visualized using UV illuminator (VilberLourmat, France). The frequency of polymorphisms and the number of bands produced by each primer were calculated individually.

### 4.8. Data Analysis

Microsoft Excel was used to analyze the morphological data. For the number of petals, flower length (cm), flower diameter (mm), number of inflorescences, number of flowers, and plant height, significant differences were evaluated using Student *t*-tests at *p* ≤ 0.05.

The SCot, ISSR and RAPD-based PCR loci were scored as present 1 or absent 0, each of which was treated as independent. Genetic diversity was identified by comparing the banding patterns of all genotypes. Polymorphism levels were estimated by dividing the polymorphic loci by the total number of scored loci. Percentage fidelity of the RAPD/SCoT was calculated from the following equation (number unique bands of RAPD-PCR/number unique bands ofSCoT-PCR × 100—total number of RAPD loci/total number of SCot loci ×100). 100 bp ladder (Invitrogen, Waltham, MA, USA) was used to estimate band size. Genetic similarities among kalanchoe genotypes were calculated according to Dice coefficient measurement [44] using IBM SPSS statistics software [45]. The clustering analysis method was used to generate the phylogeny dendrogram [46] using STATISTICA 8 software package [47].

## 5. Conclusions

This work discussed the genetic relationship among five kalanchoe genotypes through the analysis of morphological and molecular features. The information about molecular and phenotypic properties has great importance in the future selection of breeding populations, particularly for traits that possess commercial values. According to the findings, orange, violet, and yellow kalanchoe are distinct parents for breeding commercially valuable kalanchoe features. The study also concluded that SCoT and RAPD markers exhibited accurate banding pattern profiles to analyze the genetic diversity of kalanchoe genotypes as they gave a high polymorphism percentage among kalanchoe genotypes compared with ISSR marker that gave a very low polymorphism percentage.

## Figures and Tables

**Table 1:** Numbers of loci; total, monomorphic, polymorphic, and unique, generated by four out of ten primers of each RAPD, ISSR and SCot-PCR reaction in five kalanchoe genotypes, and the associated polymorphism.

PCR Type	Primer	Number of Loci	Monomorphic Loci	Polymorphic Loci	Unique Loci	Polymorphism (%)	Percentage Fidelity of the RAPD/SCoT
RAPD	OPA 2	2	1	1	0	50%	4/5 × 100− 15/38 × 100 = 80 − 39.47 = 40.53%
	OPA 7	8	2	6	2	75%	
	OPA 9	3	1	2	2	66%	
	OPA 10	2	1	1	0	50%	
Average		3.75	1.25	2.5	1	60.25%	
Total		15	5	10	4		
ISSR	ISSR-3	4	4	0	0	0%	
	ISSR-5	3	3	0	0	0%	
	ISSR-8	6	4	2	0	33%	
	ISSR-10	4	3	1	0	25%	
Average		4.25	3.5	0.75	0	**15%**	
Total		17	14	3	0		
SCoT	SCoT 3	9	3	6	3	66%	
	SCoT 11	9	5	4	1	44%	
	SCoT 13	11	4	7	1	63%	
	SCoT 14	9	4	5	0	55%	
Average		9.5	4	5.5	1.25	57%	
Total		38	16	22	5		
Total number of loci		**70**	**35**	**3** **5**	**9**		

**Table 2:** Codes and sequences of RAPD, ISSR and SCoT primers.

No.	RAPD Primers		ISSR Primers		SCoT Primers	
	Code	Sequence ′3–5′	Code	Sequence ′3–5′	Code	Sequence ′3–5′
**1**	OPA2	TGCCGAGCTG	ISSR-1	(ga) 6 gg	SCoT 2	ACCATGGCTACCACCGGC
**2**	OPA7	GAAACGGGTG	ISSR-2	(cac)3 gc	SCoT 3	ACGACATGGCGACCCACA
**3**	OPA9	GGGTAACGCC	ISSR-3	(gag) 3 gc	SCoT 4	ACCATGGCTACCACCGCA
**4**	OPA10	CTGCTGGGAC	ISSR-4	cac (tcc) 5	SCoT 5	CAATGGCTACCACTAGCG
**5**	OPA18	AGGTGACCGT	ISSR-5	tgta (ca) 7	SCoT 6	CAATGGCTACCACTACAG
**6**	OPB5	TGCGCCCTTC	ISSR-6	tac (ca) 7	SCoT 9	ACAATGGCTACCACTGCC
**7**	OPC4	CCGCATCTAC	ISSR-7	(ag) 8 t	SCoT 11	ACAATGGCTACCACTACC
**8**	OPC5	GATGACCGCC	ISSR-8	cgtc (ac) 7	SCoT 12	CAACAATGGCTACCACCG
**9**	OPC8	TGGACCGGTG	ISSR-9	tcga (ca) 7	SCoT 13	ACCATGGCTACCACGGCA
**10**	OPD5	TGAGCGGACA	ISSR-10	(ag) 8 ct	SCoT 14	ACCATGGCTACCAGCGCG

## Data Availability

Not applicable.
